# Blood Pressure Estimation Using Photoplethysmography Only: Comparison between Different Machine Learning Approaches

**DOI:** 10.1155/2018/1548647

**Published:** 2018-10-23

**Authors:** Syed Ghufran Khalid, Jufen Zhang, Fei Chen, Dingchang Zheng

**Affiliations:** ^1^Faculty of Medical Science, Anglia Ruskin University, Bishop Hall Ln, Chelmsford CM11SQ, UK; ^2^Department of Electrical and Electronic Engineering, Southern University of Science and Technology, Shenzhen 518055, China

## Abstract

**Introduction:**

Blood pressure (BP) has been a potential risk factor for cardiovascular diseases. BP measurement is one of the most useful parameters for early diagnosis, prevention, and treatment of cardiovascular diseases. At present, BP measurement mainly relies on cuff-based techniques that cause inconvenience and discomfort to users. Although some of the present prototype cuffless BP measurement techniques are able to reach overall acceptable accuracies, they require an electrocardiogram (ECG) and a photoplethysmograph (PPG) that make them unsuitable for true wearable applications. Therefore, developing a single PPG-based cuffless BP estimation algorithm with enough accuracy would be clinically and practically useful.

**Methods:**

The University of Queensland vital sign dataset (online database) was accessed to extract raw PPG signals and its corresponding reference BPs (systolic BP and diastolic BP). The online database consisted of PPG waveforms of 32 cases from whom 8133 (good quality) signal segments (5 s for each) were extracted, preprocessed, and normalised in both width and amplitude. Three most significant pulse features (pulse area, pulse rising time, and width 25%) with their corresponding reference BPs were used to train and test three machine learning algorithms (regression tree, multiple linear regression (MLR), and support vector machine (SVM)). A 10-fold cross-validation was applied to obtain overall BP estimation accuracy, separately for the three machine learning algorithms. Their estimation accuracies were further analysed separately for three clinical BP categories (normotensive, hypertensive, and hypotensive). Finally, they were compared with the ISO standard for noninvasive BP device validation (average difference no greater than 5 mmHg and SD no greater than 8 mmHg).

**Results:**

In terms of overall estimation accuracy, the regression tree achieved the best overall accuracy for SBP (mean and SD of difference: −0.1 ± 6.5 mmHg) and DBP (mean and SD of difference: −0.6 ± 5.2 mmHg). MLR and SVM achieved the overall mean difference less than 5 mmHg for both SBP and DBP, but their SD of difference was >8 mmHg. Regarding the estimation accuracy in each BP categories, only the regression tree achieved acceptable ISO standard for SBP (−1.1 ± 5.7 mmHg) and DBP (−0.03 ± 5.6 mmHg) in the normotensive category. MLR and SVM did not achieve acceptable accuracies in any BP categories.

**Conclusion:**

This study developed and compared three machine learning algorithms to estimate BPs using PPG only and revealed that the regression tree algorithm was the best approach with overall acceptable accuracy to ISO standard for BP device validation. Furthermore, this study demonstrated that the regression tree algorithm achieved acceptable measurement accuracy only in the normotensive category, suggesting that future algorithm development for BP estimation should be more specific for different BP categories.

## 1. Introduction

Blood pressure (BP) is one of the main risk factors for cardiovascular diseases. Abnormal BP has been a potent issue that causes strokes, heart attacks, and kidney failure [1]. At present, cuff-based BP measurement devices have been widely used in hospital settings to detect abnormal BP [2]. However, they are not convenient and comfortable for the users.

In the past few years, various research groups have attempted numerous techniques in order to achieve cuffless BP measurement. The key measuring principle for cuffless BP estimation is based upon the time taken by a pulse from the heart to the finger. They are known as pulse transit time (PTT) or pulse arrival time (PAT) [3–10]. Other researchers used vascular transit time (VTT) which was calculated from the time difference between photoplethysmograph (PPG) measured at the fingertip and phonocardiograph measured at the chest [11]. Cuffless BPs were also measured using the tonometry technique based on the information from multiple pressure sensors on the radial artery tree [6, 12]. Another group of researchers introduced the cuffless BP measurement technique using modified normalised pulse volume and heart rate [13]. Multiple magnetic sensors have also been used to measure pulse wave velocity (PWV) for the estimation of cuffless BP [14]. Although some of the cuffless BP devices achieved overall acceptable accuracies, the above mentioned algorithms required at least two sensors [15], making them unsuitable for true wearable applications. Therefore, developing a single PPG-based cuffless BP estimation algorithm with enough accuracy would be clinically and practically useful.

Recently, machine learning algorithms, including support vector machine (SVM), multiple linear regression (MLR), and neural networks algorithms, have been used to estimate cuffless BP. Zhang and Feng applied the SVM algorithm to waveform features that were extracted from PPG signal segments collected from the University of Queensland Vital Signs dataset [16]. Nevertheless, their study only achieved the SBP and DBP measurement accuracies of 11.6 ± 8.2 mmHg and 7.6 ± 6.7 mmHg [16]. Kawanaka et al. tested MLR algorithm with their own collected dataset. Their training data included old individuals while testing datasets gathered from young individuals [17]. Visvanathan et al. also used PPG signal features with both linear regression and SVM algorithms to estimate cuffless BP [18]. However, these studies failed to meet ISO noninvasive BP device accuracy (average difference no greater than 5 mmHg and SD no greater than 8 mmHg). Other researchers also developed a cuffless BP measurement device with acceptable accuracy in terms of mean difference (3.8 mmHg for SBP and 4.6 mmHg for DBP) accuracy, but unfortunately, their measurement techniques have not been described in detail [19]. Furthermore, in all the published studies, the measurement accuracies have not been evaluated specifically in different clinical BP categories (normotensive, hypertensive, and hypotensive).

This research aimed to develop and compare three machine learning algorithms (regression tree, MLR, and SVM) to estimate BPs only using pulse waveform features derived from good quality PPG signals. In addition, their estimation accuracy would be evaluated for three different clinical BP categories (normotensive, hypertensive, and hypotensive).

## 2. Methods

**Figure 1:**
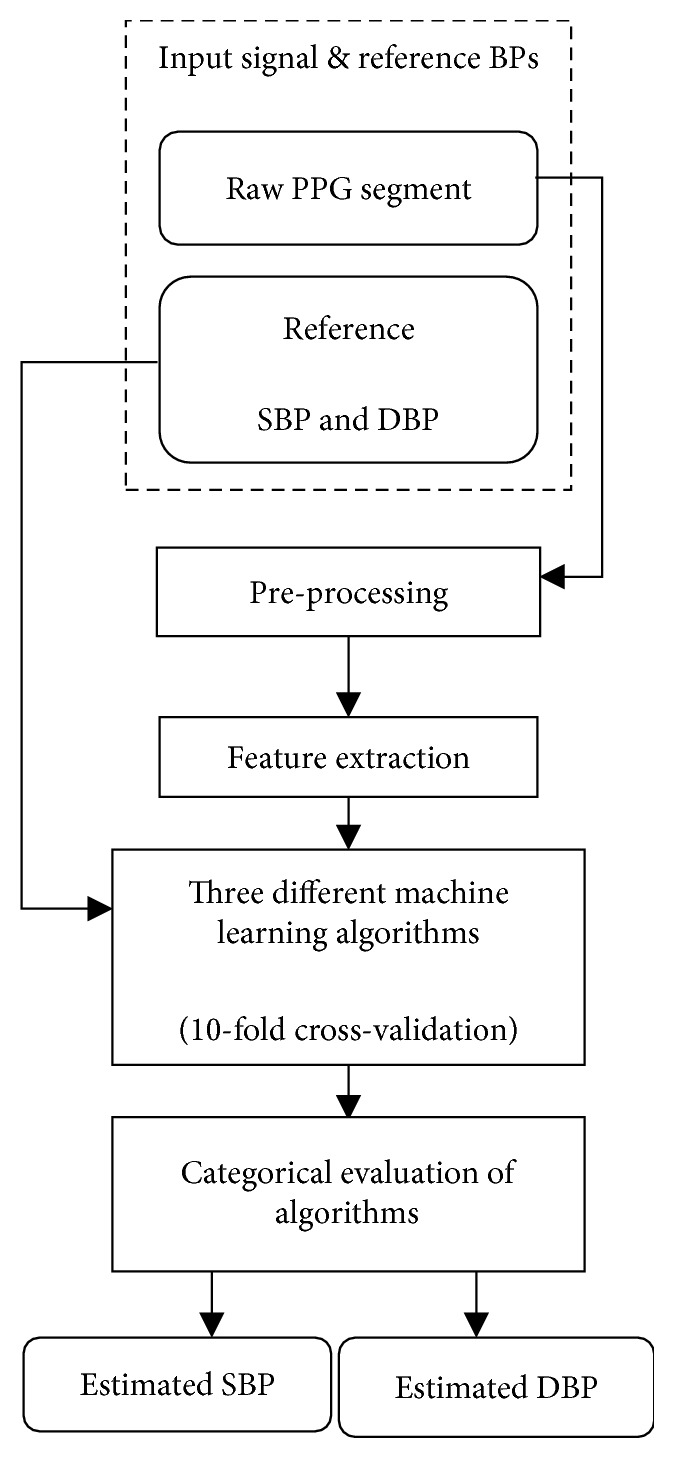
Flow diagram of research methodology.

The overall flow diagram of the proposed research methodology is presented in Figure 1, which is summarised in the following steps:

1. Extract PPG signal segments and reference BPs (SBP and DBP). Only the acceptable quality of 5 s data segments was saved.
2. Preprocess PPG signal segments, including baseline removal and PPG pulse waveform normalization.
3. Derive waveform features from preprocessed PPG signal segments.
4. Train and test with 10-fold cross-validation of three different machine learning algorithms to compare the overall estimation accuracy.
5. Evaluate estimation accuracy of the three machine learning algorithms specifically for each BP category.

### 2.1. Online Database

**Figure 2:**
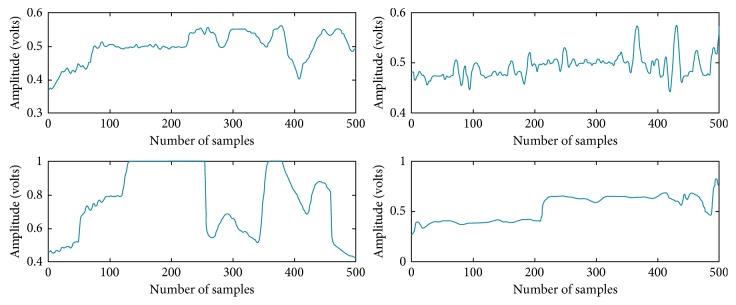
Examples of the bad quality PPG signal segments that cannot be processed and used to extract their waveform features.

The University of Queensland vital signs dataset (accessed on February 2017) was used in this study. The dataset was recorded from 32 cases in Royal Adelaide Hospital using Phillips IntelliVue MP70 and MP30 with the sampling rate of 100 Hz. The signal length from each case ranged from 13 minutes to 5 hours. Raw PPG signal waveforms with their corresponding noninvasive BP (NIBP) data were extracted [20]. The length of each extracted segment was 5 seconds. During data segmentation, a manual check was performed to avoid unacceptable quality of the PPG signal with the movement artefact and to exclude the segments without corresponding reference SBP and DBP data. The manual check was performed to ensure our machine learning models being developed did not have any interference of bad signals, allowing the BP results from different machine learning approaches to be more comparable. The number of unacceptable signal segments and the segments without reference SBP and DBP data were 9772 and 5572.  Figure 2 illustrates some examples of bad quality PPG segments.

**Figure 3:**
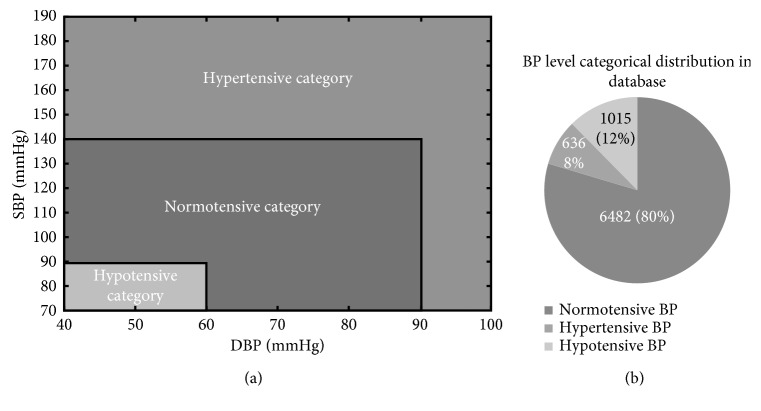
(a) BP classification chart to define the three BP categories and (b) categorical distribution of reference BPs of good quality PPGs in the database.

In total, as given in Table 1, 8133 signal segments of both good quality PPG and reference NIBP data were collected from the online database of 23617 signal segments. Next, each of the good quality segments was grouped into three different BP categories according to their reference BPs and the BP classification chart, as shown in Figure 3(a). The normotensive category included 6482 segments which were about 80% of the total good quality segments. The remaining hypertensive and hypotensive categories contained 1015 (12%) and 636 (8%), respectively, as shown in Figure 3(b). Since the BPs varied during the long period of recording, each case included variable BP segments under different BP categories, as shown in Table 1.

### 2.2. PPG Signal Preprocessing

**Figure 4:**
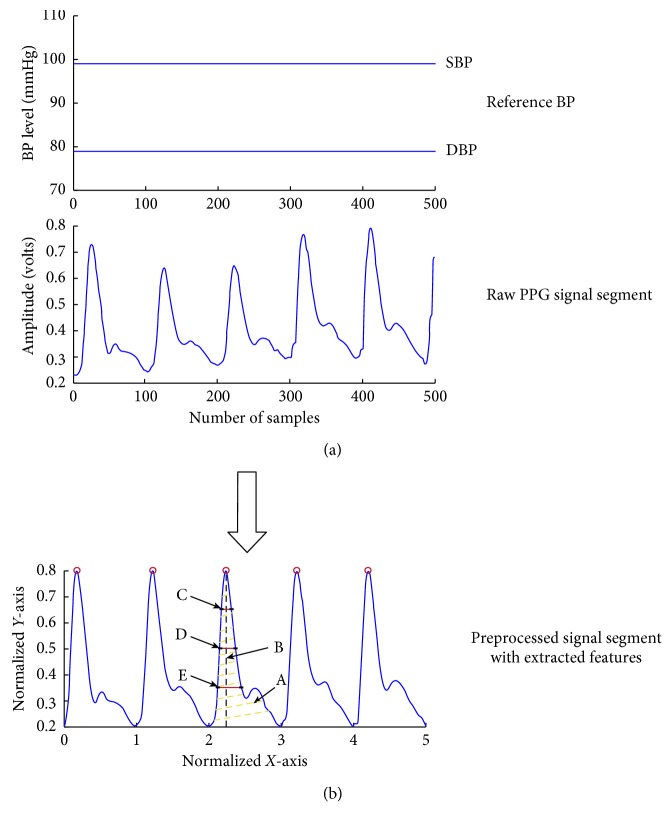
Illustration of preprocessing of raw PPG signals to normalised pulses and demonstration of extracted waveform features. (a) The two horizontal straight lines are for the reference BPs, and the middle subfigure shows a 5 s raw PPG signal segment; (b) preprocessed signal segment with extracted features indicated by alphabets (*A* *=* *pulse area*, *B* *=* *pulse rising time*, *C* *=* *width_75%*, *D* *=* *width_50%*, and *E* *=* *width_25%).*

Each PPG segment was firstly processed with a 4th order and 19 frame length Savitzky–Golay filter. This filter is a moving average filter to smooth the PPG signal. It was selected due to the advantage of sharp edge preservation [21]. Baseline wandering caused by the respiratory activity was also removed from the segments. The 2-dimensional normalization (in both width and amplitude) was then performed.  Figure 4 shows how a raw PPG segment is transformed to a normalised pulse. Since the reference NIBP was constant during the 5-second period of the segment, no further preprocessing of reference NIBP was required.

### 2.3. Features Extraction and Selection

Five different waveform features were initially extracted from each of the preprocessed PPG segments, which consisted of pulse area, pulse rising time, width 25%, width 50%, and width 75%. The “pulse area” feature of the PPG segment reflects the vascular tone changes [22]. Pulse rising time is associated with BP changes. It has been reported that it appeared earlier in younger than in older individuals [23]. Sinha et al. included this important feature in their algorithm to estimate cuffless BP [18]. The PPG pulse widths are associated with the systemic vascular resistance [24].

To select the most significant features, the multicollinearity test was applied in this study. The presence of multicollinearity among the predictor variables affects the generalizability of the algorithm, causing a high estimated mean square error of the algorithm. Variance inflation factor (VIF) as an important diagnostic tool for multicollinearity among predictors, was used to determine the presence of collinearity among predictors [25]. If VIF of a predictor is larger than 10, it indicates that the predictor is highly collinear with another predictor. The most significant features were identified with the multicollinearity test on the basis of their VIF. After the multicollinearity, width_50% and width_75% were eliminated from the training dataset due to their VIF > 10.

### 2.4. Machine Learning Algorithms to Estimate BPs

The training and testing dataset consisted of three most significant PPG waveform features (pulse area, pulse rising time, and Width_25%) from each of the 8133 PPG segments and their corresponding reference BPs (SBP and DBP). Due to the continuous nature of data, three commonly used regression-based machine learning algorithms were applied in this study as follows.

#### 2.4.1. Multiple Linear Regression (MLR)

**Figure 5:**
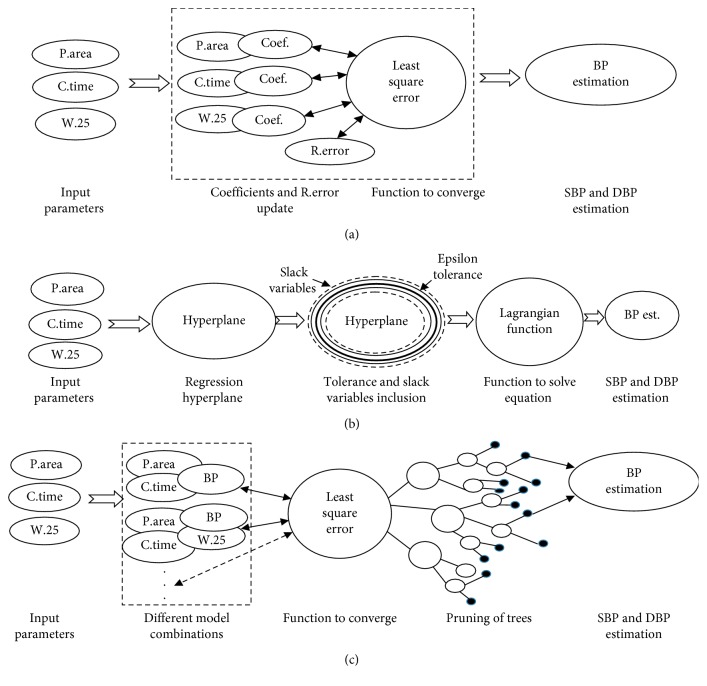
(a) Simplified flow diagrams of MLR in which coefficients and the random error were updated in each iteration to converge the least square error function. (b) Flow diagrams of SVM regression. Epsilon and slack variables surrounding the hyperplane contributed to make the dual objective formula with the Lagrangian function to solve the equation for BP estimators. (c) Simplified flow diagrams of regression tree algorithms. Different algorithm combinations were used to derive least square function, prune, and split tree in to branch nodes. Each node (small black colour-filled circles) contains an estimation result.

MLR is a type of the machine learning algorithm that has been widely used by previous researchers to estimate cuffless BP [3, 7, 26]. The algorithm started with the random selection of coefficients of the linear algorithm (*θ*_0_, *θ*_1_, *θ*_2_, and *θ*_3_). Each predictor was associated with a coefficient as shown in a virtual box in Figure 5(a). After each iteration, the coefficients and random error (*ε*, the difference between the estimated and reference BP) were updated. The least square algorithm was used to minimize the squared error as shown in Equation (1). Iterative minimization of the squared error continued until it converged when BP estimation was generated:

(1) $$\begin{array}{c} J\left(\theta_{0},\theta_{1},\theta_{2},\theta_{3}\right)=\frac{1}{m}\overset{m}{\underset{i=1}{\sum }}\left(h_{\theta }\left(x^{\left(i\right)}\right)-y^{\left(i\right)}\right),\\ h_{\theta }\left(x\right)=\left(\theta_{0}+\theta_{1}\left(\text{area}\right)+\theta_{2}\left({\text{crest}}_{\text{time}}\right)+\theta_{3}\left(\text{Width}\_25\right)\right)+\varepsilon , \end{array}$$

where *m *=* *total number of training data (90% of 8133), *ε *=* *random error, *θ*_0–3_* *= coefficients, *h*(*x*) = estimated BP, and *y* = reference BP.

#### 2.4.2. Support Vector Machine (SVM)

SVM is a nonparametric algorithm that uses kernel function. SVM regression has a similar goal as in the least square method of MLR to minimize the error function (squared error between the estimated and reference BP). However, its approach for minimizing the function is different with MLR as it uses epsilon (*ε*), and the goal is to find a function whose error was no greater than *ε*. In this study, linear epsilon SVM (*ε*-SVM) regression which is also called L1 loss was implemented. *ε*-SVM has two boundaries across the hyperplane (regression line), as shown in the line across hyperplane in Figure 5(b). However, in reality, not all residuals were laid in epsilon boundary. Therefore, slack variables (another boundary) were introduced to cover all the remaining residuals, as shown in a dashed line across hyperplane in Figure 5(b). Slack variables were added to make a dual objective. Each iteration updated the vectors existing in a dual objective, and the equation was analytically solved by Lagrangian function.

In SVM, the convergence criteria were based on the following equation:

(2) $$\begin{array}{c} \Delta =\frac{J\left(\beta \right)+L\left(\alpha \right)}{J\left(\beta \right)+1}, \end{array}$$

where *J*(*β*) is called the primal objective. *L*(*α*) is a dual objective that was solved by the Lagrangian function. The goal was to minimize the Lagrangian function to get BP estimations. Δ represents the feasibility gap. To converge the algorithm, feasibility gap should be less than the gap tolerance [27].

#### 2.4.3. Regression Tree

Regression tree algorithm is another nonparametric machine learning approach for making predictions. It is a relatively fast algorithm to train the data as compared to the SVM algorithm. It carries decisions from the root nodes to the leaf nodes. Regression trees are the binary trees, and the leaf that contains responses is in numeric form [28]. It splits the data with the best optimization criteria (that subject to tree depth (*α*); minimum leaf size (*β*)) on each predictor (pulse area, pulse rising time, and width_25%). Criterion for stopping the split to make a pure node based on the mean square error (MSE) is shown as follows:

(3) $$\begin{array}{c} \text{MSE}\left(\text{observed }\mathrm{response}\right)<\text{MSE}\left(\text{observed }\mathrm{response}\text{ from }\mathrm{all}\text{ data}\right)\times \text{tolerance}. \end{array}$$

A pure node indicates that the MSE of the observed response is less than the MSE of the observed response from all the data multiplied by the tolerance [28]. For optimization, the algorithm splits the branches of trees to minimize the prediction error as shown in Figure 5(c).

### 2.4. Tenfold Cross-Validation

In total, 8131 × 3 good quality PPG signal features and reference BPs were used to train and test the above three machine learning algorithms with 10-fold cross-validation. In each iteration, 9 folds were used to train an algorithm, and the remaining fold was used to test that algorithm. The process continued until 10 iterations were completed. In the end, there was one estimated SBP and one DBP for each of the 8133 signal segments.

### 2.5. Data Analysis to Evaluate Overall Measurement Accuracy

The three machine learning algorithms (regression tree, MLR, and SVM) were firstly evaluated in terms of overall BP estimation accuracy. After the 10-fold cross-validation of all available segments, each segment contained reference BPs (mmHg), estimated BPs (mmHg), and the difference (mmHg) between reference and estimated BP.

The averaged BPs (including both reference and estimated BPs) were calculated for each case based on all the available segments in that case. The final mean and SD of estimated BPs were then calculated for all 32 cases as an overall estimation for SBP and DBP, separately for the three machine learning algorithms. They were then compared with their reference BPs in each case to obtain overall estimation accuracy (mean difference and SD of difference).

### 2.6. Data Analysis to Evaluate Measurement Accuracy in Each BP Category

For the categorical evaluation, the estimated BPs for each of the available PPG segments in each case were separated into three groups according to their reference BP category (normotensive, hypertensive, and hypotensive). For each case, the averaged BPs were then calculated from all the available segments under each category, which were used to obtain overall BPs across all the 32 cases, separately for each BP category. Finally, the mean difference and SD of difference between the reference and estimated BPs were calculated for each BP category and plotted using the Bland-Altman method.

## 3. Results

### 3.1. Comparison of Overall BP Measurement Accuracy

**Figure 6:**
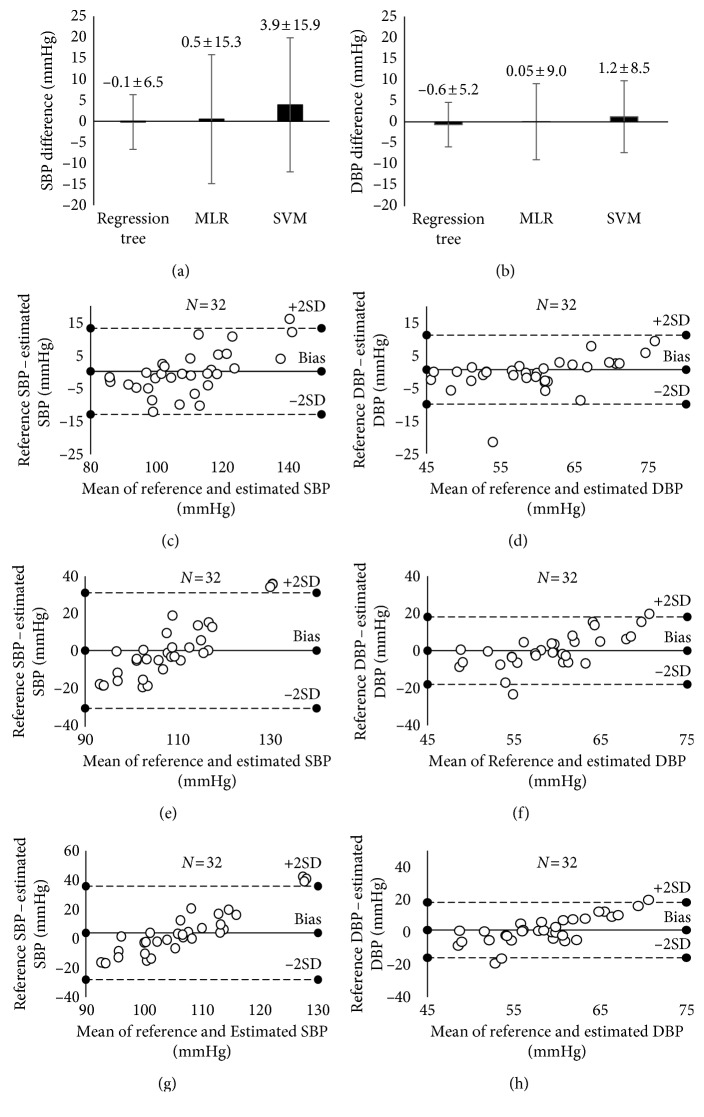
(a, b) Overall BP measurement accuracy from the 10-fold cross-validation, separately for the three machine learning algorithms; (c–h) Bland–Altman plots for the BPs estimated from the regression tree, MLR, and SVM. (c), (e), and (g) are for SBP, and (d), (f), and (h) are for DBP. MLR, multiple linear regression; SVM, support vector machine.

The overall BP measurement accuracy, as shown in Figures (6(a) and 6(b)) and Table 2, showed that the regression tree achieved the smallest mean difference of SBP (−0.1 mmHg between reference and estimated SBP) and SD of difference (6.5 mmHg) when compared with the MLR and SVM algorithms. Similarly, the regression tree achieved an acceptable mean difference (−0.6 mmHg between reference and estimated SBP) and SD of difference (5.2 mmHg) for DBP. It was also observed that only the regression tree method achieved overall acceptable accuracy to ISO standard for NIBP device validation with an average difference no greater than 5 mmHg and SD no greater than 8 mmHg. Figures (6(c)–6(h)) shows the Bland–Altman plots between the reference and estimated BPs from the three machine learning algorithms.

### 3.2. BP Measurement Accuracy under Each BP Category

**Figure 7:**
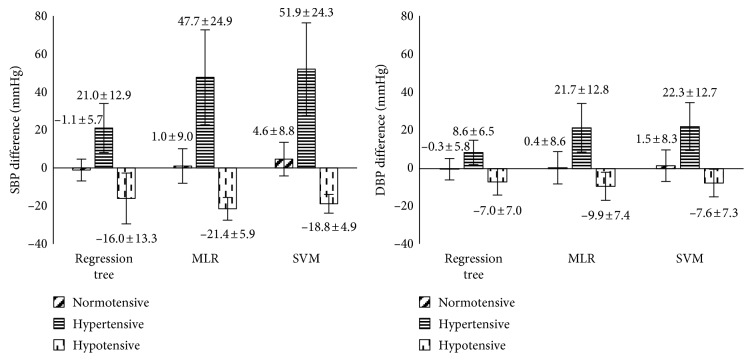
BP estimation accuracy under each BP category, separately for the three machine learning algorithms (regression tree, MLR, and SVM). The data are presented with mean BP difference ± SD of BP difference. MLR, multiple linear regression; SVM, support vector machine.

The estimation accuracies of the three machine learning algorithms under each BP category are presented in Figure 7. It can be seen that only the regression tree achieved acceptable accuracy to meet the ISO standard for device evaluation, and it was only observed in normotensive BP category. Its mean differences and SDs of difference for SBP and DBP were −1.1 ± 5.7 mmHg and −0.3 ± 5.6 mmHg. The detailed results from the regression tree for each BP category are presented in Tables 3 and 4. It can be seen that the regression tree algorithm produced higher mean differences and SD of difference under both hypertensive and hypotensive BP categories in comparison with normotensive category. It was also observed that, although the mean differences for the MLR and SVM algorithms were acceptable in the normotensive category, they did not achieve an acceptable ISO standard for device evaluation in terms of SD of difference, as shown in Figure 7.

## 4. Discussion

In this study, the overall BP estimation accuracy from three supervised machine learning algorithms (regression tree, MLR, and SVM) was compared to determine which algorithm was better to estimate cuffless BPs using PPG signals only. To prevent the selection of an overfitted algorithm, the 10-fold cross-validation was used to test the overall measurement accuracy of the algorithms. The results showed that the regression tree achieved better overall accuracy in terms of mean and SD of BP difference as required by the ISO [29].

Researchers have attempted to develop the MLR algorithm for PTT-based cuffless BP estimation [7, 30]. Although the MLR algorithm in those studies achieved acceptable measurement accuracy, their research was still susceptible to the practical issues with two sensors for the measurement. Measurements from multiple wearable sensors could cause restricted movement and discomfort to the users [31]. Another group also used the MLR algorithm with tonometry for the estimation of cuffless BP, and they succeeded to pass the ISO requirement [12], but MLR is sensitive to the outliers as shown in Figure 6(e), suggesting that MLR is probably not an ideal algorithm for BP estimation [32]. In this study, SD of BP difference was higher than the requirement of no more than 8 mmHg, and this was partially due to the presence of outliers.

The SVM algorithm has been used to estimate cuffless BP using heart sound signals, where acceptable BP measurement accuracy was achieved [33]. Similarly, in our study, the SVM algorithm was applied to PPG signal features to estimate cuffless BP. However, the SVM algorithm did not achieve acceptable accuracy with high SD of BP difference. The performance of the SVM algorithm is mostly based on the selection of the kernel. Three different kernels (linear, Gaussian, and polynomial) have been widely used [34]. In this study, the linear kernel was used to get the estimation output because the selected signal features and their corresponding BPs were in linear relationships. Zhang and Feng used the same database (University of Queensland) but with different PPG signal features to test three machine learning algorithms (MLR, neural network, and SVM). In their study, SVM achieved best measurement accuracy for SBP (11.6 ± 8.2 mmHg) and DBP (7.6 ± 6.7 mmHg), which were not up to the ISO standard [16]. Therefore, there is a need to better understand the potential reasons to improve the algorithm development.

Regression tree algorithm is robust to the noisy data and able to make a better-fitted algorithm for discrete target data [28]. Researchers used the regression tree algorithm for PTT-based cuffless BP estimation and achieved acceptable results [35]. In this study, the regression tree algorithm was among the best algorithm for BP estimation. The possible reason behind the success of regression tree is their nonvulnerability to the outliers. Another strong characteristic of this algorithm is that it also produces a well-fitted algorithm in the presence of slight nonlinearity within the data [28].

Most importantly, this study further analysed the estimation accuracy of the three machine learning algorithms under different BP categories (normotensive, hypertensive, and hypotensive) and found that most of the algorithms exhibited better accuracy in the normotensive category. Previous research only presented overall BP accuracies (overall mean of difference ± SD of difference) rather than individual categorical BP accuracies [3, 9, 36]. Some studies only included normotensive subjects [10, 17, 37]. In our study, regression tree was found with higher mean BP difference and SD of difference in hypertensive and hypotensive categories in comparison with the normotensive group. This could be caused by the low amount of data within the hypertensive and hypotensive categories of the online database. To make an accurate algorithm for each BP category, it is therefore suggested that the specific algorithm approach for different BP categories should be considered in a future study.

This study has some limitations. Firstly, manual check to determine the quality of PPG signal segments is not practical in real scenario. The development of advanced preprocessing algorithms to automatically determine signal quality is important. It is also worth investigating the effect of noise on the estimation accuracy of machine learning models. Secondly, the training and test of the three machine learning algorithms were limited to the database of the University of Queensland. It would be useful to test the algorithms in a new database. Thirdly, due to the lack of the basic clinical variables (e.g., BMI, gender, weight, and height) in the dataset, these variables were not included to train the machine learning algorithms, which may improve the measurement accuracy of some of the algorithms [12]. Finally, the BP estimation was performed on the basis of each segment and only noninvasive intermittent BPs were available to be used as reference BPs to train the algorithms. In a future study, using continuous BP as reference BPs may improve the algorithms, allowing beat-to-beat BP estimation.

## 5. Conclusions

This study developed and compared three machine learning algorithms to estimate BPs using PPG only and revealed that the regression tree algorithm was the best approach with overall acceptable measurement accuracy to the ISO standard for device validation. Furthermore, this study demonstrated that the regression tree algorithm achieved acceptable measurement accuracy only in the normotensive category, suggesting that the future algorithm development for BP estimation should be more specific for different BP categories.

## Figures and Tables

**Table 1:** The number of segments for each BP categorical groups, separately for each case.

Case	Normotensive	Hypertensive	Hypotensive	Bad quality signals	Without reference BPs	Total
Case 1	581	—	64	419	376	1440
Case 2	12	—	14	166	—	192
Case 3	1099	—	50	1400	1051	3600
Case 4	496	—	—	649	295	1440
Case 5	50	32	—	352	1726	2160
Case 6	357	56	69	158	80	720
Case 7	128	—	56	289	247	720
Case 8	248	—	—	296	152	696
Case 9	465	—	—	178	77	720
Case 10	44	1	—	195	—	240
Case 11	395	51	71	203	—	720
Case 12	312	—	392	468	268	1440
Case 13	324	—	8	223	165	720
Case 14	—	—	61	95	—	156
Case 15	—	—	12	158	—	170
Case 16	—	—	46	86	28	160
Case 17	65	—	4	70	27	166
Case 18	81	—	—	81	—	162
Case 19	40	15	—	84	20	159
Case 20	286	3	11	256	164	720
Case 21	101	0	59	119	81	360
Case 22	56	52	11	167	74	360
Case 23	22	76	—	226	—	324
Case 24	20	—	—	160	—	180
Case 25	101	57	19	468	75	720
Case 26	152	21	7	367	173	720
Case 27	98	—	—	388	101	720
Case 28	231	—	—	403	79	720
Case 29	211	—	27	480	—	720
Case 30	48	—	—	84	—	132
Case 31	315	72	34	798	221	1440
Case 32	144	200	—	286	90	720
Total	6482	636	1015	9772	5570	23617

**Table 2:** Estimated BPs (SBP and DBP) from the regression tree with their corresponding reference BPs and their difference. The results are given separately for each case.

Case	SBP (mmHg)			DBP (mmHg)		
	Reference BP	Estimated BP	Difference	Reference BP	Estimated BP	Difference
1	96.8	97.1	−0.3	49.1	49.1	0.0
2	102.1	112.0	−9.9	43.2	64.7	−21.5
3	98.7	100.7	−1.9	53.1	53.1	0.0
4	107.5	108.1	−0.6	58.2	58.6	−0.3
5	139.6	135.7	4.0	71.8	69.3	2.5
6	112.3	108.3	4.1	58.4	56.7	1.7
7	91.5	96.3	−4.8	44.4	46.8	−2.5
8	103.6	105.3	−1.8	52.1	53.1	−1.0
9	103.0	100.7	2.4	46.0	46.0	−0.1
10	118.4	107.1	11.3	51.8	50.5	1.3
11	109.9	111.0	−1.1	67.4	66.0	1.4
12	89.6	93.5	−3.9	53.0	53.2	−0.2
13	101.2	101.8	−0.6	64.4	61.5	2.9
14	84.8	86.9	−2.1	56.7	56.4	0.3
15	84.5	87.6	−3.1	49.6	52.4	−2.8
16	85.0	86.6	−1.6	56.2	57.3	−1.1
17	92.9	105.1	−12.1	57.5	59.4	−1.9
18	113.6	117.7	−4.1	59.3	62.6	−3.2
19	116.9	116.3	0.6	58.1	63.9	−5.8
20	115.2	115.7	−0.6	59.8	62.8	−3.0
21	94.4	103.0	−8.6	45.4	51.2	−5.7
22	128.3	117.7	10.7	59.7	60.1	−0.4
23	148.4	132.4	16.1	80.5	71.1	9.4
24	95.0	100.0	−5.0	61.4	70.1	−8.7
25	124.2	123.1	1.0	65.8	63.6	2.1
26	121.3	116.1	5.2	59.0	60.5	−1.6
27	124.0	118.6	5.4	71.1	68.2	2.8
28	108.3	115.0	−6.7	59.6	62.4	−2.8
29	103.2	101.6	1.6	61.3	60.3	1.0
30	108.0	118.3	−10.3	71.1	63.3	7.8
31	118.0	118.7	−0.7	72.3	69.8	2.5
32	147.2	135.1	12.0	77.4	71.6	5.8
Mean	108.9	109.1	−0.1	59.2	59.8	−0.6
SD	16.8	12.8	6.5	9.4	7.2	5.2

**Table 3:** Estimated SBP from the regression tree for each individual case under the three categories and its difference with reference SBP.

Case	Normotensive			Hypertensive			Normotensive		
	Reference SBP	Estimated SBP	Difference	Reference SBP	Estimated SBP	Difference	Reference SBP	Estimated SBP	Difference
1	98.0	97.6	0.4	—	—	—	85.7	92.4	−6.7
2	120.2	108.2	12.1	—	—	—	85.1	115.7	−30.6
3	99.5	100.7	−1.1	—	—	—	80.2	99.1	−18.9
4	107.6	108.2	−0.6	—	—	—	—	—	—
5	121.8	120.4	1.4	188.7	185.6	3.2	—	—	—
6	108.5	104.5	4.0	188.9	155.4	33.5	84.2	93.2	−9.0
7	97.3	101.6	−4.3	—	—	—	87.6	90.7	−3.1
8	104.4	105.9	−1.5	—	—	—	—	—	—
9	105.7	103.2	2.4	—	—	—	—	—	—
10	117.7	107.0	10.7	104.0	104.0	0.0	—	—	—
11	111.1	110.2	1.0	131.9	130.6	1.3	87.7	100.5	−12.8
12	103.0	103.3	−0.3	—	—	—	78.9	85.7	−6.8
13	101.7	102.2	−0.6	—	—	—	84.0	86.5	−2.5
14	—	—	—	—	—	—	84.8	87.0	−2.1
15	—	—	—	—	—	—	84.6	88.2	−3.6
16	—	—	—	—	—	—	85.0	86.6	−1.6
17	93.6	103.9	−10.3	—	—	—	82.5	124.4	−41.9
18	113.7	117.1	−3.4	—	—	—	—	—	—
19	106.3	115.9	−9.6	145.0	117.3	27.8	—	—	—
20	116.0	115.8	0.2	144.0	105.9	38.1	87.0	116.0	−29.0
21	99.9	106.3	−6.4	—	—	—	85.0	97.8	−12.8
22	116.0	111.6	4.4	150.4	124.9	25.5	87.0	117.4	−30.4
23	123.2	134.1	−11.0	155.7	131.8	23.9	—	—	—
24	95.0	97.7	−2.7	—	—	—	—	—	—
25	119.5	120.2	−0.7	147.8	127.7	20.1	83.7	125.9	−42.1
26	117.0	114.9	2.0	162.0	124.3	37.7	87.0	109.5	−22.5
27	124.0	118.6	5.4	—	—	—	—	—	—
28	106.3	115.2	−8.9	135.5	121.2	14.3	—	—	—
29	105.7	102.9	2.8	—	—	—	83.8	91.8	−8.0
30	108.0	118.3	−10.3	—	—	—	—	—	—
31	114.9	118.5	−3.6	147.0	125.0	22.0	85.2	105.7	−20.5
32	118.7	122.7	−4.0	171.6	146.0	25.6	—	—	—
Mean	109.4	110.5	−1.1	151.7	130.7	21.0	84.6	100.7	−16.0
SD	8.9	8.6	5.7	22.9	21.5	12.9	2.3	13.5	13.3

**Table 4:** Estimated DBP from the regression tree for each individual case under the three BP categories and its difference with reference DBP.

Case	Normotensive			Hypertensive			Hypotensive		
	Reference DBP	Estimated DBP	Difference	Reference DBP	Estimated DBP	Difference	Reference DBP	Estimated DBP	Difference
1	49.8	49.3	0.5	—	—	—	45.7	46.8	−1.0
2	43.0	64.3	−21.3	—	—	—	44.0	65.1	−21.1
3	54.5	53.3	1.2	—	—	—	48.4	50.0	−1.6
4	59.0	58.5	0.5	—	—	—	—	—	—
5	67.0	65.6	1.4	79.1	79.1	0.0	—	—	—
6	60.5	57.2	3.3	88.0	74.0	14.0	41.6	46.8	−5.2
7	49.3	48.4	0.9	—	—	—	43.1	45.6	−2.5
8	53.0	53.5	−0.5	—	—	—	—	—	—
9	49.3	48.2	1.1	—	—	—	—	—	—
10	51.9	49.9	2.0	54.7	54.7	0.0	—	—	—
11	69.9	66.3	3.6	93.0	80.3	12.7	49.2	54.3	−5.0
12	63.2	60.2	3.0	—	—	—	46.7	47.7	−1.0
13	64.6	61.5	3.0	—	—	—	56.0	60.2	−4.2
14	—	—	—	—	—	—	57.6	56.7	0.9
15	—	—	—	—	—	—	54.3	54.3	0.1
16	—	—	—	—	—	—	56.2	57.3	−1.1
17	58.3	59.8	−1.6	—	—	—	45.5	52.8	−7.3
18	59.7	62.2	−2.5	—	—	—	—	—	—
19	51.6	64.6	−13.0	77.0	60.9	16.1	—	—	—
20	60.3	62.5	−2.2	76.0	77.4	−1.4	50.0	65.8	−15.8
21	45.4	51.6	−6.2	—	—	—	45.7	50.6	−4.9
22	63.1	58.9	4.2	62.5	61.3	1.2	40.0	62.5	−22.5
23	74.1	69.1	5.0	82.3	71.7	10.6	—	—	—
24	61.5	67.3	−5.8	—	—	—	—	—	—
25	63.9	62.0	1.9	73.2	66.1	7.1	51.7	63.0	−11.3
26	57.4	60.2	−2.8	75.0	60.2	14.8	53.0	67.0	−14.0
27	71.1	68.2	2.8	—	—	—	—	—	—
28	60.7	63.2	−2.5	87.5	72.3	15.2	—	—	—
29	65.8	61.7	4.1	—	—	—	52.3	57.5	−5.2
30	71.1	63.3	7.8	—	—	—	—	—	—
31	70.8	68.5	2.3	87.4	76.0	11.5	57.4	68.3	−10.9
32	66.6	65.5	1.1	88.0	76.8	11.2	—	—	—
Mean	59.8	60.1	−0.3	78.7	70.0	8.6	49.3	56.4	−7.0
SD	8.2	6.3	5.6	10.9	8.4	6.5	5.4	7.4	7.0

## Data Availability

The database used in this study is available to access via the link: https://outbox.eait.uq.edu.au/uqdliu3/uqvitalsignsdataset/index.html.
